# Influences of Intermittent Preventive Treatment and Persistent Multiclonal *Plasmodium falciparum* Infections on Clinical Malaria Risk

**DOI:** 10.1371/journal.pone.0013649

**Published:** 2010-10-27

**Authors:** Anne Liljander, Daniel Chandramohan, Margaret Kweku, Daniel Olsson, Scott M. Montgomery, Brian Greenwood, Anna Färnert

**Affiliations:** 1 Infectious Diseases Unit, Department of Medicine Solna, Karolinska Institutet, Stockholm, Sweden; 2 Infectious and Tropical Diseases Department, London School of Hygiene and Tropical Medicine, London, United Kingdom; 3 Ghana Health Service, University of Ghana, Accra, Ghana; 4 Medical Statistics Unit, Department of Learning Informatics Management and Ethics, Karolinska Institutet, Stockholm, Sweden; 5 Clinical Epidemiology Unit, Department of Medicine Solna, Karolinska Institutet, Stockholm, Sweden; 6 Clinical Epidemiology and Biostatistics Unit, Örebro University Hospital, Örebro, Sweden; 7 Department of Primary Care and Social Medicine, Charing Cross Hospital, Imperial College, London, United Kingdom; Walter and Eliza Hall Institute of Medical Research, Australia

## Abstract

**Background:**

Intermittent preventive treatment (IPT) of malaria involves administration of curative doses of antimalarials at specified time points to vulnerable populations in endemic areas, regardless whether a subject is known to be infected. The effect of this new intervention on the development and maintenance of protective immunity needs further understanding. We have investigated how seasonal IPT affects the genetic diversity of *Plasmodium falciparum* infections and the risk of subsequent clinical malaria.

**Material and Methods:**

The study included 2227 Ghanaian children (3–59 months) who were given sulphadoxine-pyrimethamine (SP) bimonthly, artesunate plus amodiaquine (AS+AQ) monthly or bimonthly, or placebo monthly for six months spanning the malaria transmission season. Blood samples collected at three post-interventional surveys were analysed by genotyping of the polymorphic *merozoite surface protein 2* gene. Malaria morbidity and anaemia was monitored during 12 months follow-up.

**Results:**

Monthly IPT with AS+AQ resulted in a marked reduction in number of concurrent clones and only children parasite negative just after the intervention period developed clinical malaria during follow-up. In the placebo group, children without parasites as well as those infected with ≥2 clones had a reduced risk of subsequent malaria. The bimonthly SP or AS+AQ groups had similar number of clones as placebo after intervention; however, diversity and parasite negativity did not predict the risk of malaria. An interaction effect showed that multiclonal infections were only associated with protection in children without intermittent treatment.

**Conclusion:**

Molecular typing revealed effects of the intervention not detected by ordinary microscopy. Effective seasonal IPT temporarily reduced the prevalence and genetic diversity of *P. falciparum* infections. The reduced risk of malaria in children with multiclonal infections only seen in untreated children suggests that persistence of antigenically diverse *P. falciparum* infections is important for the maintenance of protective malaria immunity in high transmission settings.

## Introduction

Malaria is one of the most severe infectious diseases. Despite recent reports of decreasing incidence in some areas, infection by *Plasmodium falciparum* remains a major threat to health and economic development in Sub-Saharan Africa [1]. Children under five years of age and pregnant women are at highest risk of disease and death. Control strategies targeting these groups are, therefore, of high priority. A new strategy to control malaria is intermittent preventive treatment (IPT), which involves administration of therapeutic doses of anti-malarial drugs at specified time points regardless whether a subject is known to be infected. IPT is a modification of previously used chemoprophylaxis which was not considered to be sustainable due to logistic difficulties, concerns over the emergence of drug resistant parasites and impaired development of malaria immunity. The advantage of intermittent treatment over sustained chemoprophylaxis is reduced drug exposure. The intervention is implemented in pregnant women (IPTp), reducing anaemia in the mother and increasing birth weight of the child [2]. Several studies of IPT in infants (IPTi) with sulphadoxine-pyrimethamine (SP) given at the age of 2, 3 and 9 months alongside the extended program on immunization have shown a reduction in the incidence of clinical malaria and anaemia by about 30% [3] and IPTi is now recommended for implementation in areas with high burden of malaria and low SP resistance [4].

IPTi is likely to be most effective in areas with continuous high malaria transmission. However, in many areas of sub-Saharan Africa, especially those with seasonal transmission, the main burden of malaria is in older children. An alternative approach in these areas is administration of IPT to children (IPTc) during the high transmission season. Seasonal IPT reduced the incidence of clinical malaria by 17–86% depending on antimalarial drug and dosage regime [5], [6].

Some rebound in malaria morbidity was reported following sustained chemoprophylaxis [7], [8] and some IPT studies have reported increased incidence of clinical malaria or anaemia in sub-groups of subjects when the intervention was stopped [9], [10], [11]. In spite of these reports, a meta-analysis of all the published trials of IPTi did not find any evidence that IPT leads to rebound of malaria in infants [3]. Whether intermittent treatment affects immunity towards malaria when given as seasonal intervention and in older children who have acquired partial protection is however unclear.

In high transmission areas, immunity to malaria develops gradually following repeated exposure and *P. falciparum* infections are often composed of multiple genetically distinct clones. The ability to control diverse infections may be a key feature of protective immunity. The number of clones correlates with transmission intensity and age [12], [13], [14], [15]. In areas of intense transmission, asymptomatic multiclonal infections have been associated with a reduced risk of subsequent clinical malaria in partially immune children [16], [17], whereas in young children and in moderate to low transmission settings, a high number of clones appears to be a risk factor for disease [18], [19], [20]. The number of clones thus appears to be a marker of exposure and immunity. Persistence of low density infections composed of antigenically diverse parasites may *per se* be important to maintain protection in areas of intense transmission. For these reasons we have investigated how intermittent treatment affects subsequent *P. falciparum* infections and risk of clinical malaria and anaemia within a seasonal IPT trial in Ghanaian children [10].

## Materials and Methods

### Study site

A randomized, placebo-controlled IPTc trial was conducted in Hohoe district, Ghana during 2005 to 2006 [10]. Malaria is endemic in the area with peak transmission following the rainy seasons in April to July and September to November, with an estimated transmission intensity of ∼65 infective bites/person/year [10]. Written consent was obtained from the caretakers of participating children. Ethical approval was granted by the Ghana Health Services/Ministry of Health, London School of Hygiene and Tropical Medicine Ethics Committee and the Regional Ethical Review Board in Stockholm, Sweden.

### Study outline

**Figure 1:**
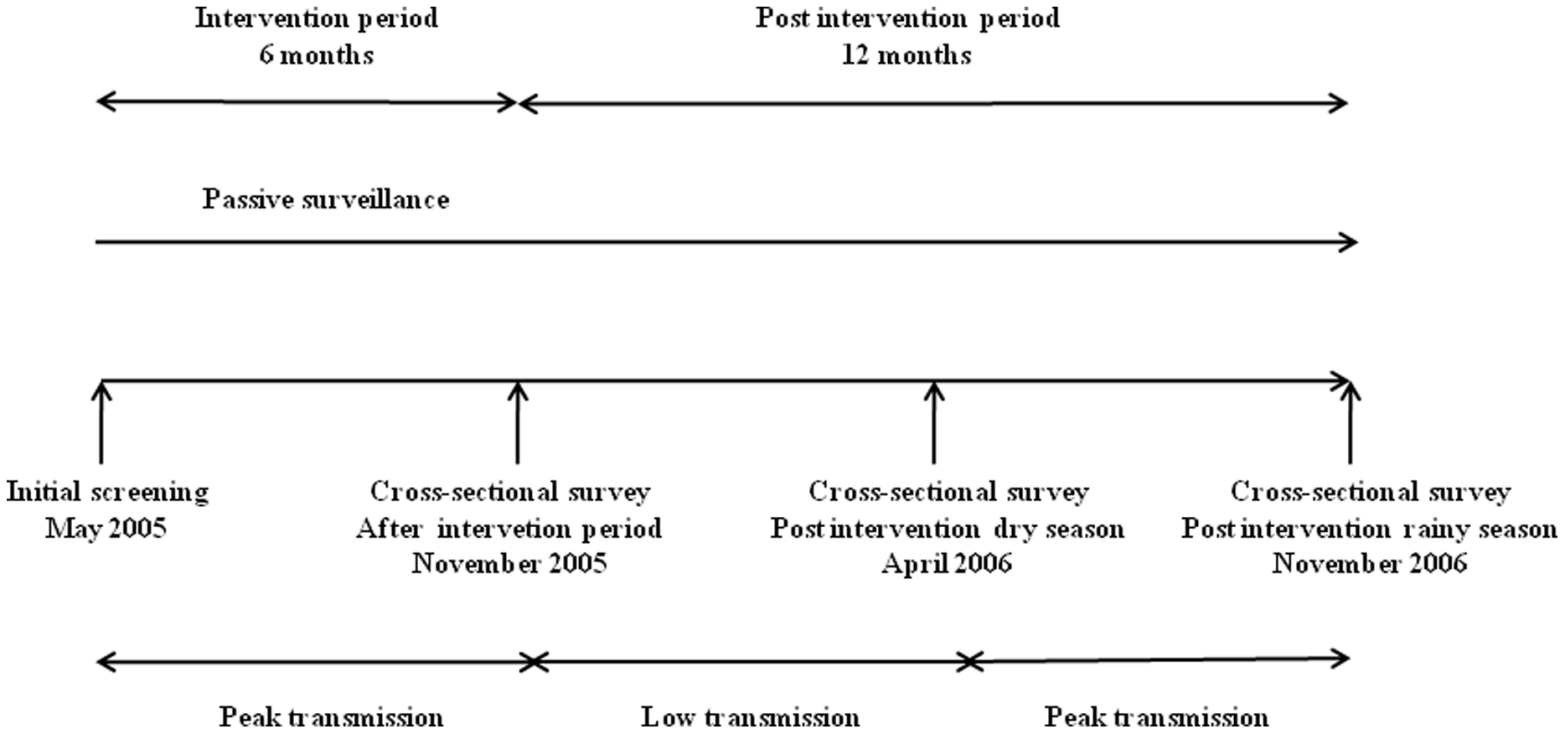
Study outline. Intermittent preventive treatment or placebo was given over a six month-period spanning peak malaria transmission season, followed by a 12- month post intervention surveillance period. Cross-sectional surveys were performed 1 month (November 2005), 6 months (April 2006) and 12 months (November 2006) after the intervention was stopped. Blood films and filter paper samples were collected at these three time points.

In total 2227 of the 2451 participating children (aged 3 to 59 months), provided comprehensive data required for this study. The IPTc trial has been described in detail elsewhere [10]. In brief, enrolled children were allocated to either treatment regimen; SP bimonthly (every second month), AS +AQ bimonthly, AS +AQ monthly, or a placebo monthly, given over a six month period of intense malaria transmission (Figure 1). A drug simulating placebo was given on alternate months in the bimonthly groups. During the intervention period, children were visited weekly at their homes for health assessment. The parents were advised to take the child to the health facility for examination and treatment if the child had a history of fever of vomiting within the past 48 hours. Three cross-sectional surveys were performed during the 12 months follow-up in addition to passive surveillance for clinical malaria and anaemia at the study hospital.

### Clinical case definitions

A clinical episode of malaria was defined as fever (axillary temperature ≥37.5° C or reported fever within the past 48 hours) together with any *P. falciparum* parasiteamia detected by microscopy. A second definition of malaria was fever (defined as above) and a peripheral parasiteamia of >7000 parasites/µl blood, a local threshold with high specificity for clinical malaria [10]. All malaria episodes were treated with oral quinine for seven days according to national guidelines. Anaemia was defined as a haemoglobin (Hb) concentration less than 8 g/dl blood [10].

### Cross sectional surveys and sample collection

Initial screening for eligibility was performed one month before administration of the first IPT dose (May 2005). After the six-month intervention period (June-October 2005), three cross-sectional surveys were performed: 1) at the end of the intervention period (November 2005); 2) at the end of following dry season, (April 2006); and 3) at the end of the following rainy season (November 2006) (Figure 1). Axillary temperature and Hb concentration were measured at all surveys. Blood films and filter paper (Grade 541, Whatman) samples were collected at the three cross-sectional surveys, however not at the initial screening. Parasites were counted by light microscopy against 200 leukocytes and converted to parasites/µl following the assumption of 8000 leukocytes/µl blood. Filter papers were stored in individual plastic bags at room temperature.

### Genotyping of *msp2*

Filter paper samples from microscopy positive children collected at the three cross-sectional surveys were all analyzed by *msp2* genotyping. DNA was extracted from whole blood spots on filter paper using ABI Prism 6100 Nucleic Acid PrepStation (Applied Biosystems). *P. falciparum* infections were characterized by genotyping of the *merozoite surface protein 2 (msp2)* gene using fluorescent PCR and capillary electrophoresis (CE) [21]. In brief, in the primary PCR, oligonucleotide primers span the outer region of *msp2* (block 3) followed by separate, nested reactions with fluorescently labeled primers distinguishing the respective allelic types of *msp2*, FC27 and IC. CE was performed on a 3730 DNA sequencer using GeneMapper® Software (v 4.0) (both Applied Biosystems). A cut off of 300 relative fluorescent units was used to distinguish true alleles from non-specific low artefact peaks [21].

### Statistical methods

Data analysis was performed using R (v 2.9.0) and SPSS (v 17). Comparisons of parasite prevalence between treatment groups were assessed by Chi-square tests. Associations between the number of clones and parasite densities were analysed by Spearman rank. The presence of high number of clones i.e. ≥3 were compared between the groups by Fisher's exact test. When used as explanatory variable, the number of *msp2* clones was categorised to avoid assumptions about linearity as follows: 0 i.e. negative by microscopy, 1 and ≥2 clones. Age was grouped into five categories (3–11, 12–23, 24–35, 36–47 and 48–59 months) and parasite densities into quartiles. Association between number of clones and treatment group, age, parasite densities, sex and clinical malaria during intervention (yes/no) was assessed by logistic regression (with clones as outcome comparing 1 with ≥2 clones). Association between number of clones and anaemia (Hb<8.0 g/dl) (with anaemia yes/no as outcome) at the respective surveys was analyzed by logistic regression.

The prospective risk of clinical malaria was assessed by Cox regression analysis based on time to first or only clinical episode during the 12-month follow-up. The analysis included children who participated in the first post-intervention survey, with one or two subsequent samples. Analysis was performed for all children and then per separate treatment group. The data on clones, age and parasite densities was categorised as described above. To identify strictly asymptomatic individuals, children with clinical episode during the period 28 days before until 7 days after survey were excluded from the survival analyses since number of clones can be affected by clinical episode or recent treatment [22], [23]. Hazard ratios (HR) were assessed in a model including number of *msp2* clones (0, 1 and ≥2), sex, age, owning a bednet, treatment group and clinical malaria (yes/no) during the intervention period. Both clinical definitions of malaria (see above) were used in the risk assessments. When the more strict definition of malaria (fever and >7000 parasites/µl) was used, 28 days were subtracted from the time at risk if children had been treated with quinine due to a clinical episode with fever and <7000 parasites/µl. The proportional hazards assumption was assessed using tests and graphical diagnostics based on scaled Schoenfeld residuals. Unadjusted Kaplan Meier survival plots with time to a first clinical episode of malaria were made for all children and for the individual treatment groups, respectively.

## Results

### Parasite prevalence post intervention

At the first survey after the intervention was stopped, the prevalence of *P. falciparum* infection by microscopy was lower (5.2%) in children who had received monthly AS+AQ treatment compared to children given placebo (19.8%) (*p<0.05*, *Chi-square*, Table 1). There was however no difference in parasite prevalence in the bimonthly SP or AS+AQ group compared to the placebo group. Six months post-intervention, at the end of the following dry season, the prevalence was low in all groups although relatively higher in children who had received IPT (Table 1). At the following rainy season, 12 months post-intervention, parasite prevalence was similar in all groups. There were no differences in parasite densities between the groups except for the SP group in which densities were higher at the two first surveys (Table 1).

**Table 1:** Parasitological findings at three cross-sectional surveys following a 6 months intervention period with different IPT regimes.

Characteristics	Placebo		SP bimonthly		AS+AQ bimonthly		AS+AQ monthly	
	All	Asympt^a^	All	Asympt^a^	All	Asympt^a^	All	Asympt^a^
**After the intervention period (n)**	591	579	555	541	502	476	579	573
Any parasiteamia^b^ (%)	19.8	19.0	17.5	16.1	20.5	18.3	5.2*	4.9*
Parasitaemia^b^>7000/µl (%)	6.9	6.7	7.9	6.8	8.2	6.3	1.6*	1.6*
Parasite density(geometric mean, (range))	2516(40–68440)	2465(40–68440)	4754(80–180800)	4014(80–92800)	3290(80–213240)	2517(80–213240)	1889(80–115320)	2010(80–115320)
*msp2* diversity (median, (range))	2 (1–8)	2 (1–8)	2 (1–5)	2 (1–5)	2 (1–6)	2 (1–6)	2 (1–2)	2 (1–2)
>1 *msp2* clone (%)	70.6	70.1	67.4	70.1	60.5	60.6	66.7	69.2
≥3 msp2 clones (%)	36.3	36.1	32.6	32.5	29.8	28.2	0	0
**Post intervention dry season (n)**	481	476	442	436	403	400	463	456
Any parasitaemia^b^ (%)	6.7	6.1	10.6*	9.9*	10.0	9.5	11.2*	10.3*
Parasitaemia^b^>7000/µl (%)	2.1	1.5	4.5*	4.1*	2.5	2.5	3.2	2.6
Parasite density(geometric mean, (range))	2112(120–121600)	1619(120–89600)	4529(240–162240)	4719(240–162240)	1776(120–140000)	1825(120–140000)	2926(80–168800)	2615(80–168800)
*msp2* diversity (median, (range))	2 (1–6)	2 (1–6)	2 (1–4)	2 (1–4)	2 (1–6)	2 (1–6)	2 (1–5)	2 (1–5)
>1 *msp2* clone (%)	50.0	50.0	55.0	54.1	69.0	67.9	54.3	53.7
≥3 msp2 clones (%)	29.2	31.8	25.0	24.3	24.3	25.0	23.9	22.0
**Post intervention rainy season (n)**	448	434	401	392	363	357	430	419
Any parasitaemia^b^ (%)	37.3	35.5	36.4	35.5	38.0	37.3	41.2	39.9
Parasitaemia^b^ >7000/µl (%)	8.9	8.3	7.5	6.9	8.8	8.1	10.2	8.8
Parasite density(geometric mean, (range))	2249(40–172480)	2231(40–172480)	2187(40–162240)	2003(40–162240)	2399(40–169240)	2214(40–141360)	2223(40–332960)	1995(40–332960)
*msp2* diversity (median, (range))	2 (1–8)	2 (1–7)	2 (1–6)	2 (1–6)	2 (1–6)	2 (1–6)	2 (1–7)	2 (1–7)
>1 *msp2* clone (%)	74.8	75.2	72.9	72.3	74.5	74.7	71.7	70.6
≥3 msp2 clones (%)	43.7	44.0	44.9	44.6	44.1	43.4	44.1	43.7

These parasitological data have been presented previously [10]. The prevalence figures presented here differ slightly from those in the previous publication because it was not possible to obtain data on parasite genotype for all the children in the study due to missing samples.

a Excluding children with clinical malaria at survey, 28 days before and 7 days after.

b Determined by microscopy.

* compared to the placebo group p<0.05 (Chi-square).

### *Msp2* diversity post intervention

**Figure 2:**
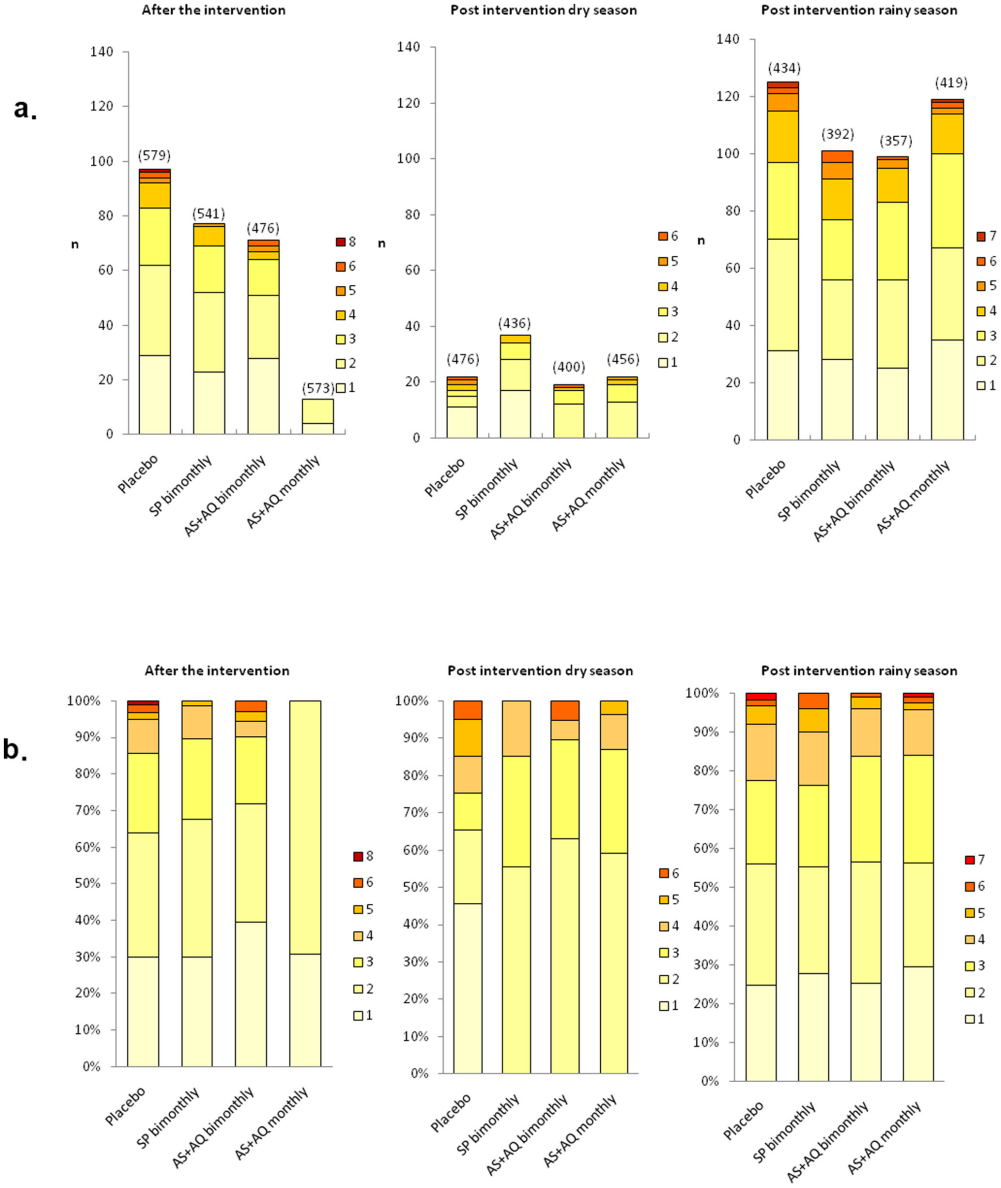
a) Number (n) and b) proportion (%) of PCR positive asymptomatic children infected with different number of *msp2* genotypes at the respective surveys; 1 month after the intervention, at the following dry season 6 months post-intervention, and at the end of the following rainy season 12 months post-intervention. The number within brackets in Figure 2a represents the total number of children in the respective groups.

One month after the intervention at the first survey, multiclonal infections (≥2 clones), were detected in 60.6–70.1% of the PCR positive samples from asymptomatic children. Children in the AS+AQ monthly group were infected with 1–2 clones (Figure 2), a significantly higher proportion of children with 3 or more clones were found in the placebo group where up to 8 clones were detected (*p = 0.009*, Fisher's exact test). No such differences were detected comparing the bimonthly groups to placebo. There was a weak correlation between number of clones and parasite densities using continuous data r = 0.20 (*p = 0.043*, *Spearman rank*). Age was associated with diversity only in the placebo group where children older than 11 months were more likely to be infected with ≥2 clones than younger children [OR 7.73, 95% CI 1.49–60.50], a difference that persisted after adjusting for parasite density and owning a bednet. Six and 12 months after the intervention, the number of clones was equally high in all groups (Figure 2).

### *Msp2* diversity and anaemia

Children with parasites were more likely to be anaemic than children without parasites [OR 2.65, 95% CI 1.91–3.68, adjusted for age, clinical episode during intervention and treatment group]. Multiple clones did not increase the likelihood of aneamia compared to single clone infection [OR 1.67, 95% CI 0.87–3.36; adjusted as above].

### *Msp2* diversity and risk of clinical malaria

During the intervention period, children receiving active treatment, especially with monthly AS+AQ, experienced fewer episodes of malaria than children given placebo [10]. During the 12 months follow-up 286 episodes were recorded in 213 children; 57 in the placebo group; 82 in the SP bimonthly group; 64 in the AS+AQ bimonthly group and 83 in the AS+AQ monthly group. Episodes with high density parasitaemia (>7000 parasites/µl) were reported in 185 episodes; 32 in the placebo group; 52 in the SP bimonthly group; 44 in the AS+AQ bimonthly group and 57 in the AS+AQ monthly group. Within the AS+AQ monthly group, only children who were parasite negative at the first survey after the intervention period developed clinical malaria during follow up. The risk of clinical malaria during follow-up was significantly higher among children given IPT with monthly AS+AQ compared to children given placebo [HR 1.55, 95% CI 1.05–2.27] while there was a tendency towards an increased risk among children in the SP bimonthly group [HR 1.36 95% CI 0.94–2.10] and the AS+AQ bimonthly group [HR 1.20 95% CI 0.78–1.83]. Similar results were seen using the more strict definition of malaria (>7000 parasites/µl).

Univariate time to event analysis including all asymptomatic children (n = 1856), showed that multiclonal infections were, compared to single clone infections, associated with a reduced risk of subsequent malaria using both clinical definitions (Table 2). The risk of malaria decreased with age and was highest in children who received AS+AQ monthly, and were <11 months when they received their first IPT dose. Parasite status (positive or negative), parasite density, sex, owning a bednet and clinical malaria during the intervention period were not associated with prospective malaria risk.

**Table 2:** Risk for subsequent clinical malaria in asymptomatic children (n = 1856) during the 12 months follow up after the intervention.

	All episodes			High parasite density episodes(>7000 parasites/µl)		
	HRunadjusted(95% CI)	HRadjusted^a^(95% CI)	HRadjusted^b^(95% CI)	HRunadjusted(95% CI)	HRadjusted^a^(95% CI)	HRadjusted^b^(95% CI)
Total						
*msp 2 clones*						
0	0.66(0.37–1.19)	0.67(0.37–1.20)	0.62(0.34–1.12)	0.61(0.31–1.20)	0.61(0.31–1.21)	0.56(0.28–1.11)
1*	1.00	1.00	1.00	1.00	1.00	1.00
≥2	**0.39**(0.17–0.91)	**0.43**(0.19–0.99)	**0.43**(0.19–0.99)	**0.32**(0.11–0.90)	0.36(0.13–1.01)	0.35(0.13–1.00)
Placebo						
*msp 2 clones*						
0	**0.36**(0.15–0.85)	**0.30**(0.12–0.73)	NA	**0.34**(0.19–0.99)	0.31(0.10–1.002)	NA
1*	1.00	1.00	1.00	1.00	1.00	1.00
≥2	**0.07**(0.0084–0.58)	**0.066**(0.0078–0.56)	NA	**0.11**(0.012–0.99)	0.17(0.01–1.14)	NA
SP bimonthly						
*msp 2 clones*						
0	1.26(0.31–5.20)	1.30(0.31–5.38)	NA	0.87(0.21–3.59)	0.93(0.23–3.76)	NA
1*	1.00	1.00	1.00	1.00	1.00	1.00
≥2	1.11(0.22–5.72)	1.23(0.24–6.43)	NA	0.44(0.06–3.06)	0.50(0.07–3.41)	NA
AS+AQ bimonthly						
*msp 2 clones*						
0	0.53(0.19–1.51)	0.53(0.19–1.49)	NA	0.51(0.16–1.66)	0.51(0.15–1.71)	NA
1*	1.00	1.00	1.00	1.00	1.00	1.00
≥2	0.58(0.14–2.31)	0.57(0.14–2.30)	NA	0.59(0.12–2.86)	0.58(0.11–2.98)	NA

The AS+AQ monthly group is not included in the table since no parasite positive children developed a clinical episode during follow up.

* reference group.

a adjusted for age.

b adjusted for age and treatment group.

NA not applicable.

No significant deviations from the proportional hazards assumptions were found.

**Figure 3:**
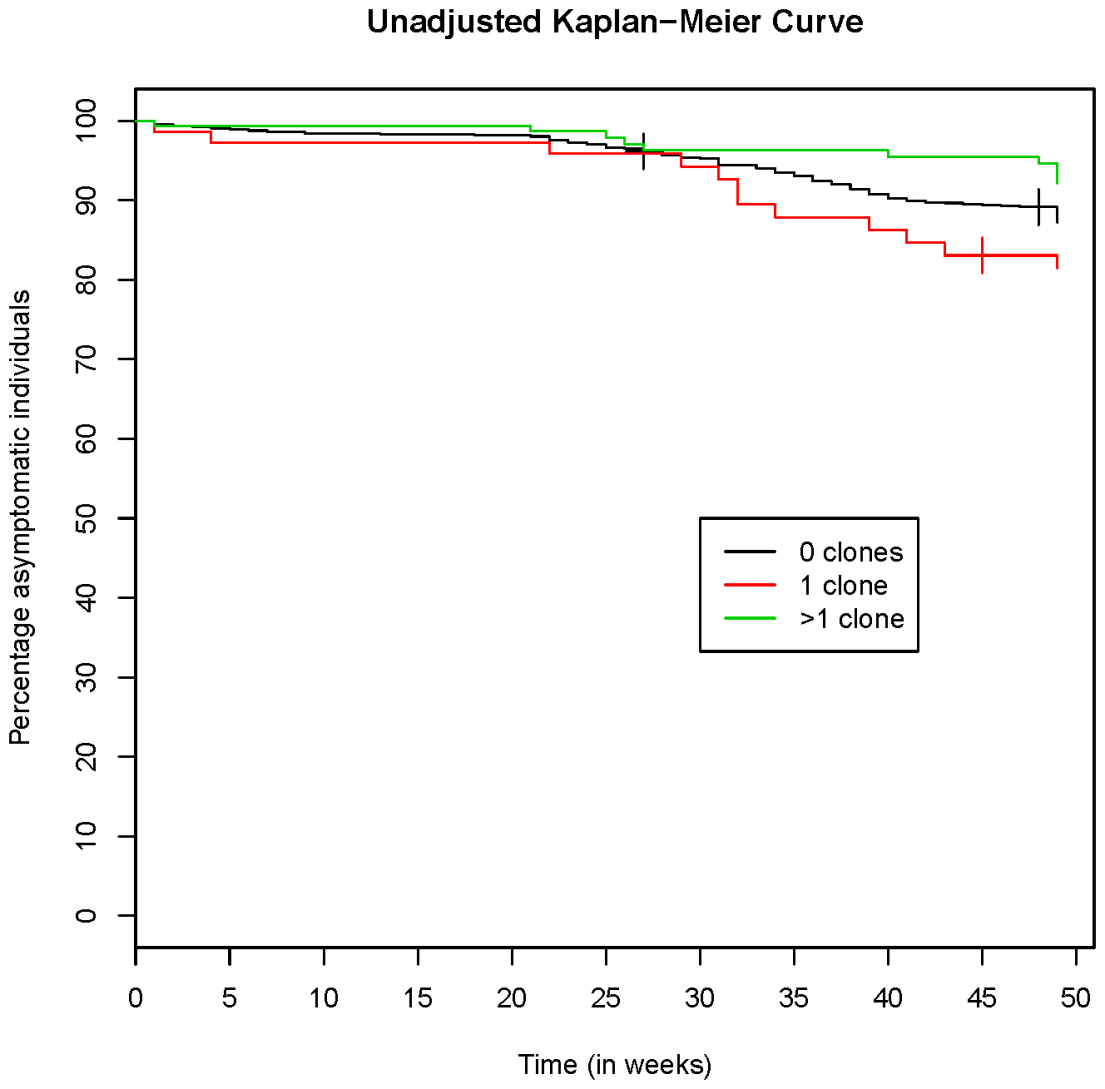
Kaplan Meier estimate (unadjusted) of time to subsequent clinical episode (fever and any *P. falciparum*) in 1856 asymptomatic children.

**Figure 4:**
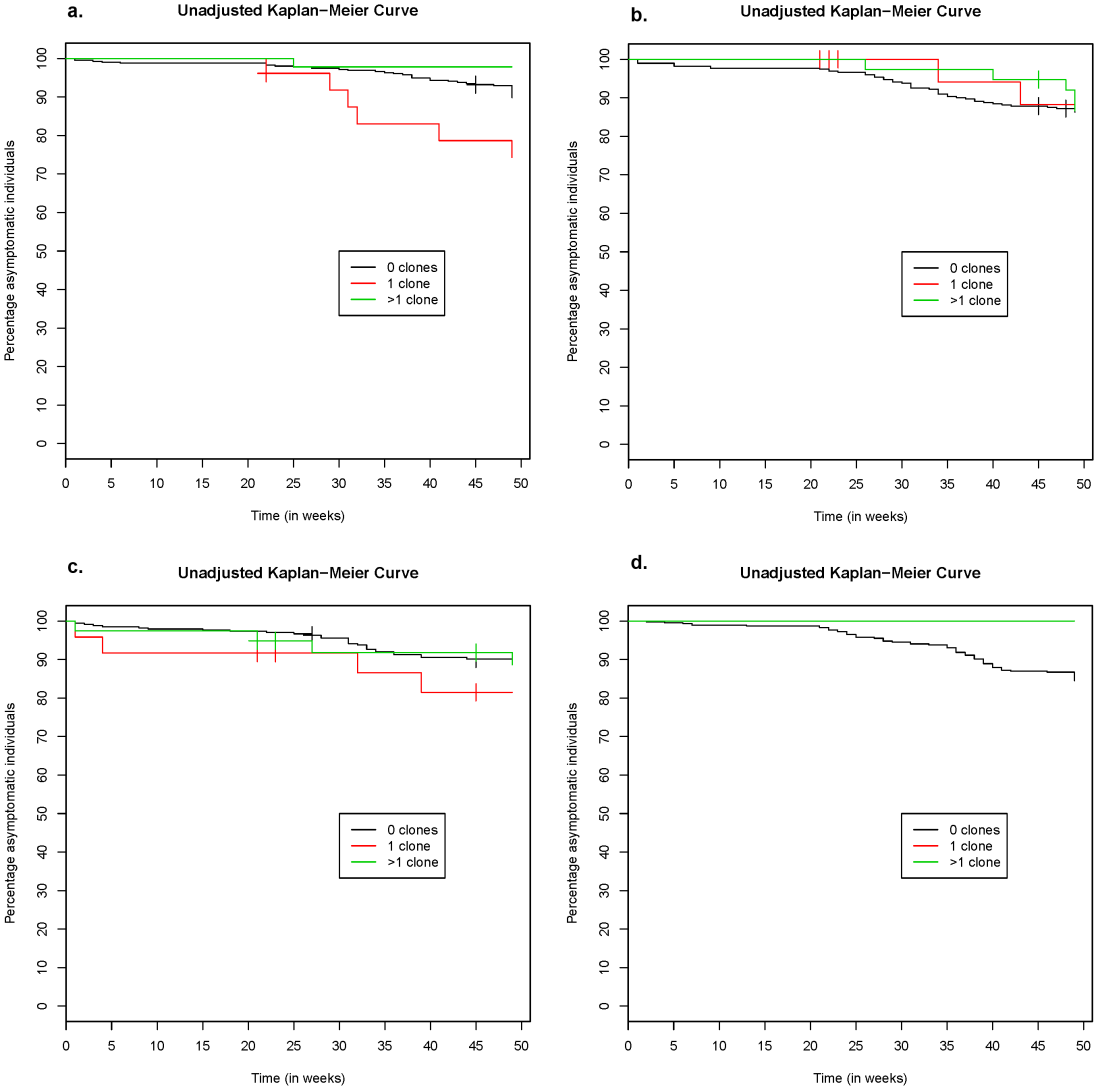
Kaplan Meier estimate (unadjusted) of time to subsequent clinical episode (fever and any *P. falciparum*) in 1856 asymptomatic children; a) placebo, b) SP bimonthly c) AS+AQ bimonthly and d) AS+AQ monthly. In the AS+AQ monthly group no children with parasites at the survey after ended intervention developed clinical malaria.

In a multivariate analysis including all asymptomatic children and adjusting for age and treatment group, the decreased risk of disease in children with multiclonal infections at baseline remained significant for both malaria definitions (Table 2/Figure 3). The association with protection was significant in the placebo group (Table 2/Figure 4A). Moreover, parasite negative children also had a decreased risk of disease compared to those with a single clone. A similar, non-significant trend was seen in the AS+AQ bimonthly group (Table 2/Figure 4C), whereas no such association was found in the SP group (Table 2/Figure 4B). In the AS+AQ monthly group, analysis regarding number of clones was not possible since none of the children with parasites at baseline developed clinical malaria during follow up (Figure 4D).

The non-significant results in the bimonthly treatment groups could have been due to the relatively few parasite positive children. An analysis investigating the interaction between treatment/placebo and protective effect of ≥2 clones was therefore conducted. In an analysis comparing groups with similar diversity after ended intervention i.e. placebo and the bimonthly IPT groups (SP and AS+AQ) an interaction between infection diversity and treatment showed that the protective effect of having ≥2 clones was significantly higher in children with placebo compared to children who had received bimonthly IPT [HR 0.092, 95% CI 0.009–0.980] The point estimates indicated that the protective effect of having ≥2 clones was relatively strong, reducing the risk by a factor of ten.

## Discussion

Intermittent treatment administered during the intense malaria transmission season affected the diversity of *P. falciparum* infections, defined by *msp2* genotyping, and its relation to the subsequent risk of clinical malaria in these children in Hohoe District in Ghana. During the 6 months intervention period, the incidence of clinical malaria and anaemia was substantially reduced [10]. Here, we present genotyping and clinical data from the 12-months follow-up after ended intervention.

With the most intense regime of monthly AS+AQ, the prevalence and diversity of *P. falciparum* infections was clearly reduced in the first survey one month after the intervention, with detection of only 1–2 clones compared to up to 6–8 clones in the other groups. This reduction was, however, only temporary and there was no difference between the groups 6 and 12 months later. Interestingly, there was a distinct difference depending on whether AS+AQ had been administered monthly (6 courses) or bimonthly (3 courses), with the last effective course one and two months before the first post-intervention survey respectively. After just one additional month, there was no difference in the number of infecting clones between the treatment and the placebo group, suggesting that multiclonal infections are rapidly accumulated in this setting.

In the monthly AS+AQ group, only children who were parasite negative at the first survey after intervention developed clinical malaria during follow-up. In the placebo group parasite negative children were at lower risk of subsequent malaria, whereas no significant risk was detected in the bimonthly groups. These parasite negative children might both represent a group with lower exposure as well as children with efficient immunity. Notably, this reduced risk was only seen in the untreated children.

When assessing whether the number of clones in asymptomatic *P. falciparum* infections predicted the subsequent risk of malaria, infections with ≥2 clones were associated with a reduced risk in the placebo group, representing the natural condition in this setting. Interestingly, children in the bimonthly IPT groups were infected with similar number of clones as children receiving placebo, and although there was a tendency in the AS+AQ bimonthly group, clones did not confer protection in neither of these two groups. The protective effect of multiclonal infections in the placebo group was, indeed, relatively strong reducing the risk by a factor of ten compared to the bimonthly IPT groups. This suggests that it is not only the number of clones present at a single time point, but infections over a preceding period that influence malaria immunity.

Asymptomatic multiclonal *P. falciparum* infections have previously been associated with a reduced subsequent risk of clinical malaria in high transmission areas [16], [17], [24], [25]. The opposite was, however, found in low transmission settings, suggesting an exposure dependent component [18], [19], [20]. Considering the short half-life of malaria specific antibody responses [26] we have hypothesized that multiclonal infections *per se* are important in stimulating protective immunity. Reduced levels of anti-*P. falciparum* IgG antibodies have been reported after sustained chemoprophylaxis [27] as well as after single-dose SP treatment within an IPTi trial [28]. Parasite prevalence was not associated with subsequent risk of clinical malaria in the current study; instead a reduced risk was associated with diversity of infections among untreated children. This supports the notion that persistent infections composed of antigenically different clones are important for protection.

The effect on malaria risk was most pronounced at the beginning of the following rainy season when the number of clinical episodes increased (Figure 3). The low parasite prevalence at the dry season survey, however, precluded this as baseline for prospective risk analysis. Although parasite prevalence was low, more than 50% of the infections detected at the dry season survey were composed of multiple clones. The ability among untreated children to control new infections as the transmission season starts might be due to low-level multiclonal infections that persisted throughout the dry season [29], [30], while previously treated children were more susceptible to disease when infected with novel parasite clones [12]. This is supported here by the finding that parasite negativity conferred protection only in children in the placebo group and not in the treatment groups.

A limitation of the current study is that blood samples were not collected before IPT was given. Such samples would have further elucidated the effect of IPT on infection diversity in the respective treatment groups and would have provided information on whether children attain the same diversity after a six months intervention period, i.e. reflecting tolerance to certain levels of diversity. Moreover, parasite prevalence and infection diversity is likely to have been underestimated since only filter paper samples from microscopy positive children were available for genotyping. Analyses of all children by PCR would have allowed for a more correct and in depth analyses of parasite positivity and diversity since the method generally detects a larger proportion of parasitized individuals in endemic settings compared to microscopy. This would also have given the opportunity to identify continuously parasite negative individuals who could be regarded as unexposed and excluded in a separate risk analysis.

Children who received placebo experienced more clinical episodes of malaria during the intervention period [10]. Quinine, the drug used for treatment, has a short half-life and would not be expected to have any prophylactic effect. There was no difference in the number of clones after intervention whether the children had been treated or not during the six months intervention so different levels of prior treatment are not likely to have been a confounding factor.

The parasitological failure rate was 2.3% for AS+AQ and 6.8% for SP, and the re-infection rates at day 28 was 16% for AS+AQ and 19% for SP in the original IPTc study [10], suggesting that the drugs were efficacious. The relatively low drug resistance in the area is therefore not likely to have had a substantial effect on the diversity of infections or malaria morbidity.

Seasonal IPT reduced the prevalence of anemia by 30–45%, but with no prolonged effect beyond the intervention period [10]. Parasite prevalence was associated with anaemia after ended intervention; however multiclonal infections were not associated with increased likelihood of being anaemic. This suggests that malaria anaemia is more likely influenced by high parasite loads than by the genetic diversity of infections.

The risk of clinical malaria during follow up was increased in infants (3–11 months) given monthly AS+AQ, and there was a tendency in this direction in the bimonthly treatment groups. A similar rebound in morbidity in infants given IPT was suggested in another high transmission setting in Ghana [9]. A meta-analysis of IPTi studies did not show a rebound in malaria morbidity after ended intervention [3]. The concept of IPTi with antimalarial treatment given at the time of routine immunization the first year of life is however different from the seasonal IPT approach in the present study where drugs are given repeatedly during the intense transmission season. The effect of IPT in infants is likely to be mainly prophylactic, preventing rather than clearing present infections. In older children, with partial immunity and often asymptomatic infections, the clearance of parasites might also be important. The reduced exposure to multiclonal infections achieved by seasonal IPT might affect immunity and explain the rebound in morbidity seen after IPT in some studies. Current recommendations for IPTi with SP state that the intervention should be implemented only in areas with moderate to high transmission [4]. Seasonal IPT might indeed have different effects in different transmission settings and age groups.

In conclusion, our findings show that seasonal IPT in children under five affects the diversity of *P. falciparum* infections. The number of clones and parasite negativity predicted a lower risk of malaria only in previously untreated children, which suggests that continuous exposure and persistent infections are important for maintenance of malaria immunity. Genotyping of *P. falciparum* infections was a useful tool to more in depth study the effect of different drug regimes for seasonal IPT on malaria exposure and morbidity.
